# Efficacy of Luseogliflozin Added to Semaglutide in Patients With Metabolic Dysfunction‐Associated Steatohepatitis and Type 2 Diabetes Mellitus: A Randomised, Open‐Label Trial

**DOI:** 10.1111/dom.71233

**Published:** 2026-08-17

**Authors:** Teruki Miyake, Osamu Yoshida, Shinya Furukawa, Masashi Hirooka, Masanori Abe, Yoshio Tokumoto, Ayumi Kanamoto, Takao Watanabe, Ryo Yano, Yuki Okazaki, Atsushi Yukimoto, Yoshiko Nakamura, Eiji Takeshita, Teru Kumagi, Yoichi Hiasa

**Affiliations:** ^1^ Department of Lifestyle‐Related Medicine and Endocrinology Ehime University Graduate School of Medicine Toon Ehime Japan; ^2^ Total Medical Support Center Ehime University Hospital Toon Ehime Japan; ^3^ Center for Data Science Ehime University Toon Ehime Japan; ^4^ Department of Gastroenterology and Metabology Ehime University Graduate School of Medicine Toon Ehime Japan; ^5^ Health Service Center Ehime University Matsuyama Ehime Japan; ^6^ Department of Community and General Medicine Ehime University Graduate School of Medicine Toon Ehime Japan; ^7^ Department of Inflammatory Bowel Diseases and Therapeutics Ehime University Graduate School of Medicine Toon Ehime Japan; ^8^ Post Graduate Medical Education Center Ehime University Graduate School of Medicine Toon Ehime Japan

**Keywords:** antidiabetic drug, clinical trial, diabetes complications, fatty liver disease, GLP‐1 analogue, SGLT2 inhibitor

## Abstract

**Aims:**

Type 2 diabetes drives the progression of metabolic dysfunction‐associated steatohepatitis (MASH). We evaluated whether antidiabetic therapy, specifically adding sodium–glucose cotransporter 2 (SGLT2) inhibitor to a glucagon‐like peptide‐1 receptor agonist (GLP‐1RA), improves liver histology in adults with biopsy‐proven MASH and type 2 diabetes.

**Materials and Methods:**

In this 52‐week, open‐label, randomised, parallel‐group trial in Japan, adults with biopsy‐confirmed MASH and type 2 diabetes received semaglutide plus luseogliflozin or semaglutide alone at antidiabetic doses. Paired liver biopsies at baseline and Week 52 were evaluated by blinded pathologists. Primary histological endpoints included resolution of MASH without worsening fibrosis, ≥ 1‐point improvement in the non‐alcoholic fatty liver disease (NAFLD) activity score without worsening fibrosis and ≥ 1‐stage improvement in fibrosis without worsening MASH.

**Results:**

Sixty participants were randomised (combination, *n* = 24; monotherapy, *n* = 36). At Week 52, all histological endpoints numerically favoured combination therapy. Resolution of MASH without worsening fibrosis occurred in 34.9% versus 19.4%, ≥ 1‐point improvement in the NAFLD activity score (NAS) in 75.5% versus 55.6% and ≥ 1‐stage fibrosis improvement in 26.9% versus 13.9%. In the pre‐specified per‐protocol analysis, ≥ 1‐point improvement in the NAS was nominally significant. Combination therapy reduced body weight, glycated haemoglobin levels, aminotransferase levels and FibroScan‐derived liver stiffness.

**Conclusions:**

In the pre‐specified full analysis set, combination therapy at antidiabetic doses did not significantly improve pre‐specified histological endpoints compared with monotherapy. In the pre‐specified per‐protocol analysis, a nominal improvement in the NAS was observed. Combination therapy improved body weight, glycemic control, aminotransferase levels and liver stiffness, with no new safety concerns identified.

**Trial Registration:** UMIN Clinical Trials Registry (UMIN‐CTR): UMIN000045003; Japan Registry of Clinical Trials (jRCT): jRCTs061210009

## 1 Introduction

Metabolic dysfunction‐associated steatotic liver disease (MASLD) has replaced the term non‐alcoholic fatty liver disease (NAFLD), reflecting a shift towards a cardiometabolic disease construct that integrates metabolic risk factors into diagnosis and risk stratification [1, 2]. The spectrum of MASLD includes metabolic dysfunction‐associated steatohepatitis (MASH) with progressive fibrosis, which is strongly linked to adverse hepatic outcomes as well as systemic complications [2]. Type 2 diabetes mellitus is highly prevalent among patients with MASLD/MASH and is associated with greater disease severity and accelerated fibrosis progression; fibrosis stage is a major determinant of long‐term prognosis [3, 4, 5]. Therefore, effective and scalable treatment strategies for patients with biopsy‐confirmed MASH and concomitant type 2 diabetes mellitus are crucial.

Lifestyle modification and weight reduction remain foundational therapies; however, sustained weight loss is difficult to achieve in routine clinical practice [2, 6]. Although pioglitazone and other agents have shown histological benefits in non‐alcoholic steatohepatitis (NASH)/MASH, concerns regarding their long‐term safety and broad clinical applicability persist [7]. Incretin‐based therapies and sodium–glucose cotransporter 2 (SGLT2) inhibitors, cornerstones of type 2 diabetes mellitus management, have garnered attention for their potential hepatic benefits alongside established cardiometabolic protection [2, 8, 9, 10, 11, 12, 13]. Glucagon‐like peptide‐1 receptor agonists (GLP‐1RAs) have demonstrated improvements in steatohepatitis activity in randomised trials, including histological responses to semaglutide [8, 9, 10]. Japanese biopsy‐based studies have reported histological improvements with SGLT2 inhibitors [11, 12]. Recent meta‐analyses further support the idea that GLP‐1RAs and SGLT2 inhibitors improve liver‐related biomarkers and steatosis‐related measures, although histological effects remain inconsistent [13, 14].

Despite these advances, evidence pertaining to the benefits of combination therapy, such as adding an SGLT2 inhibitor to GLP‐1RA therapy, for biopsy‐proven MASH remains limited. Most available data are derived from retrospective or real‐world observational studies or post hoc analyses of clinical trials and primarily rely on metabolic outcomes, non‐invasive indices or imaging‐based assessments rather than paired liver biopsies, including studies in MASLD and type 2 diabetes mellitus populations [15]. In patients with type 2 diabetes mellitus who are at high risk of MASH progression, it is important to determine whether histological improvement can be achieved using doses approved for the treatment of type 2 diabetes mellitus, without escalating to obesity‐ or MASH‐specific regimens. Semaglutide recently became the first GLP‐1RA to receive US Food and Drug Administration (FDA) accelerated approval for noncirrhotic MASH with moderate‐to‐advanced fibrosis, underscoring the expanding role of incretin‐based therapy in MASH management [16].

Therefore, we conducted and evaluated the safety of a 52‐week, randomised, parallel‐group trial in adults with biopsy‐proven MASH and type 2 diabetes mellitus to determine whether adding an SGLT2 inhibitor to GLP‐1RA therapy, at doses approved for type 2 diabetes mellitus in Japan, improves pre‐specified co‐primary histological endpoints at Week 52 compared with GLP‐1RA monotherapy [17].

## 2 Materials and Methods

### 2.1 Participants

Eligible participants were adults with type 2 diabetes mellitus and histologically confirmed MASH based on a liver biopsy performed after obtaining informed consent (screening biopsy). Histology was assessed using the NASH Clinical Research Network scoring system. Key histological inclusion criteria required a NAFLD activity score (NAS) ≥ 4 (range, 4–8) and fibrosis stages 1–4, assuming compensated cirrhosis. Major exclusion criteria were other chronic liver diseases, decompensated cirrhosis, excessive alcohol intake and contraindications to GLP‐1RA or SGLT2 inhibitor therapy (the full criteria are provided in the published protocol) [17]. This study was designed and initiated before the introduction of the MASLD nomenclature. At the time of study initiation, participants were enrolled using the NAFLD definition; however, all enrolled participants had biopsy‐confirmed steatohepatitis and type 2 diabetes mellitus, fulfilling the current diagnostic criteria for MASLD.

### 2.2 Study Design and Oversight

This investigator‐initiated, open‐label, randomised, parallel‐group trial compared GLP‐1RA and SGLT2 inhibitor combination therapy with GLP‐1RA monotherapy in adults with biopsy‐proven MASH and type 2 diabetes mellitus. The 52‐week trial was conducted at Ehime University Hospital, Japan (August 2021–June 2024), and the overall design has been published previously [17]. The study protocol was in accordance with the Declaration of Helsinki and approved by the Institutional Ethics Committee of Ehime University Hospital (approval number: N21EC001; approved on 7 May 2021). Written informed consent was obtained from all participants, and the privacy rights of the participants were observed.

### 2.3 Randomisation and Interventions

Participants were randomly assigned to the combination therapy or monotherapy groups using a minimisation algorithm (UMIN INDICE) with stratification by baseline fibrosis stage (Stages 1–3 vs. 4) and NAS (4–6 vs. 7–8). Allocation approximated a 1:1 ratio, although some numerical imbalance occurred because of probabilistic allocation. Randomisation was implemented centrally using UMIN INDICE with allocation concealment.

Participants in the combination group received semaglutide plus luseogliflozin, whereas those in the monotherapy group received semaglutide alone. Semaglutide was initiated at 0.25 mg once weekly and increased to a target maintenance dose of 0.5 mg once weekly after 4 weeks, in accordance with the approved label in Japan. All participants reached the 0.5‐mg dose at least once during the study; however, dose reduction to 0.25 mg was permitted in cases of intolerance, and one participant remained on the lower dose thereafter. Luseogliflozin was administered orally at 2.5 mg once daily in the morning, either before or after breakfast. Dose escalation to 5 mg once daily was permitted at the discretion of the treating physician when glycemic control or metabolic response was considered insufficient; this occurred in nine participants in the combination group. Chronic systemic corticosteroid therapy and concomitant use of other SGLT2 inhibitors and GLP‐1RA were prohibited throughout the study. Initiation or dose modification of pioglitazone or vitamin E was not permitted during the trial period. Although protocol‐allowed adjustments to other background antidiabetic medications were permitted when clinically indicated, no participants initiated, discontinued or modified background antidiabetic therapy after randomisation.

### 2.4 Study Visits and Assessments

Assessments were conducted at baseline and every 13 weeks thereafter (Weeks 13, 26, 39 and 52). At each visit, body weight, vital signs and adverse events were recorded. Laboratory tests included glycated haemoglobin (HbA1c), fasting plasma glucose, liver enzymes (aspartate aminotransferase [AST], alanine aminotransferase [ALT] and γ‐glutamyl transferase), renal function, lipid profile and other routine haematology and chemistry tests. Body composition was measured using a bioelectrical impedance analyser (InBody 720; Biospace Corporation Limited, Seoul, Korea).

### 2.5 Liver Biopsy and Histological Outcomes

Liver biopsies were obtained at baseline and Week 52 under laparoscopic or ultrasound guidance. Specimens were processed using haematoxylin–eosin and silver staining and assessed by two experienced hepatology pathologists blinded to the treatment assignment and clinical data. Baseline specimens were assessed prospectively before randomisation to confirm histological eligibility and to obtain the histological variables used in the allocation procedure. Subsequent biopsy specimens were assessed as they became available, and the pathologists were therefore not completely blinded to the biopsy sequence. In cases of discordant scoring, a consensus meeting was held to determine the final histological score. Fibrosis stage and NAS components (steatosis, lobular inflammation and hepatocellular ballooning) were scored according to the NASH Clinical Research Network system [18].

Three pre‐specified primary histological endpoints were evaluated at Week 52: (1) resolution of MASH without worsening of fibrosis, (2) ≥ 1‐point improvement in NAS without worsening of fibrosis and (3) ≥ 1‐stage improvement in fibrosis without worsening of MASH. Worsening of fibrosis was defined as an increase of ≥ 1 fibrosis stage from baseline. Worsening of MASH was defined as an increase of ≥ 1 point in either the lobular inflammation score or the hepatocellular ballooning score compared with baseline.

### 2.6 FibroScan Assessments

Transient elastography (FibroScan; Echosens, Paris, France) was performed at baseline and Week 52 to assess liver stiffness (kPa) and the controlled attenuation parameter (CAP; dB/m). Since probe selection was not standardised and XL‐probe data were limited, primary elastography analyses were restricted to participants with valid M‐probe measurements at both time points. Two participants in each treatment group lacked evaluable paired M‐probe measurements at Week 52 and were therefore excluded from the longitudinal FibroScan analyses. Technically unsuccessful or unreliable measurements were treated as missing.

### 2.7 Safety

Safety was assessed in all randomised participants who received at least one dose of study medication (safety analysis set). Adverse events were recorded at each visit, coded and summarised by treatment group. Serious adverse events, death and treatment discontinuation due to adverse events were also documented.

### 2.8 Statistical Analysis

The sample size was determined a priori as described in the published protocol [17]. Briefly, assuming a clinically meaningful difference in MASH resolution rates between groups, 29 participants per arm were required to achieve 80% power at a two‐sided α level of 0.05. To allow for potential dropouts and unevaluable biopsies, 60 participants (30 per group) were planned for enrollment.

Efficacy analyses followed a pre‐specified statistical analysis plan. The full analysis set (FAS) included all randomised participants and was analysed according to the assigned treatment, while the per‐protocol set (PPS) comprised participants with evaluable Week‐52 liver biopsy data.

For the primary histological endpoints, between‐group comparisons were performed using the Cochran–Mantel–Haenszel method, adjusted for randomisation stratification factors (baseline fibrosis stage and NAS categories). Effect estimates are presented as adjusted risk differences (percentage points) with 95% confidence intervals (CIs). Missing Week‐52 histological outcomes were handled using multiple imputations, with imputed datasets combined using Rubin's rules. A pre‐specified per‐protocol analysis restricted to participants with evaluable Week‐52 liver biopsy specimens was also conducted.

Key secondary continuous outcomes at Week 52 (percent change in body weight, change in HbA1c [percentage points], change in liver enzymes and percent change in FibroScan [Echosens] measurements) were analysed using analysis of covariance models, adjusting for baseline values, treatment group and stratification factors, where appropriate. Secondary outcomes were analysed using available data without imputation unless otherwise specified. Longitudinal changes through Week 52 were evaluated using mixed models for repeated measures with fixed effects for treatment, visit, and treatment‐by‐visit interactions and an unstructured covariance matrix.

For graphical presentation, time‐course plots of body weight, HbA1c, AST and ALT levels were generated using observed data from the PPS, with values presented as means ± standard errors at each pre‐specified visit (baseline and Weeks 13, 26, 39 and 52). All tests were two sided. Formal adjustment for multiplicity was not specified in the statistical analysis plan; therefore, *p* values are presented as nominal throughout the manuscript.

Additionally, an exploratory post hoc analysis was performed within the combination therapy group to compare participants who maintained luseogliflozin at 2.5 mg with those whose dose was escalated to 5 mg. Continuous and categorical variables were compared using the Wilcoxon rank‐sum and Fisher's exact tests, respectively. Because dose escalation was not randomised and was determined according to treatment response during follow‐up, these comparisons were considered exploratory and potentially subject to confounding by indication.

Analyses were performed using SAS software, version 9.4 (SAS Institute Inc., Cary, NC, USA).

## 3 Results

### 3.1 Participants

Of the 70 participants assessed for eligibility and who provided informed consent, all underwent baseline liver biopsy for eligibility assessment; 10 were excluded because their biopsy findings did not meet the histological inclusion criteria. The remaining 60 participants were randomised: 24 to combination therapy with a GLP‐1RA plus an SGLT2 inhibitor and 36 to GLP‐1RA monotherapy (Figure 1). The FAS (intention‐to‐treat) and safety analysis sets included all 60 randomised participants (*n* = 60). Week‐52 liver biopsy data were available for 58 participants (22 in the combination group and 36 in the monotherapy group); two participants in the combination group declined a repeat biopsy due to pain experienced during the baseline procedure.

**FIGURE 1:**
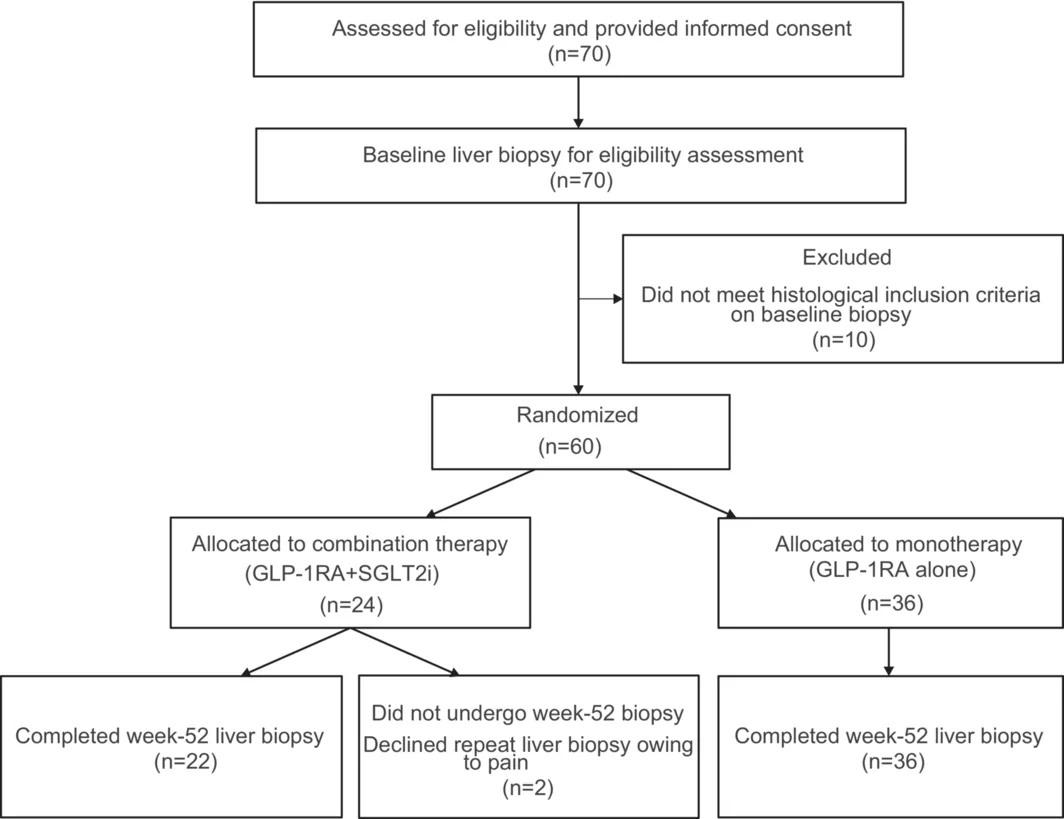
Enrollment, randomisation and follow‐up. A total of 70 participants were assessed for eligibility, provided informed consent and underwent baseline liver biopsy; 10 were excluded because the biopsy findings did not meet the histological inclusion criteria. Sixty participants were randomised to combination therapy with a glucagon‐like peptide‐1 receptor agonist (GLP‐1RA) plus a sodium–glucose cotransporter 2 inhibitor (*n* = 24) or GLP‐1RA monotherapy (*n* = 36). The full analysis set (intention‐to‐treat) and safety analysis set included all randomised participants (*n* = 60). Two participants in the combination group declined a repeat liver biopsy due to pain during the baseline biopsy and did not undergo the Week‐52 biopsy; all remaining participants completed the Week‐52 liver biopsy assessment. The per‐protocol set included 58 participants with evaluable Week‐52 liver biopsy data (combination therapy group: *n* = 22; monotherapy group: *n* = 36).

### 3.2 Baseline Characteristics

Baseline characteristics were balanced between groups (Table 1). The mean age was 56.0 ± 12.3 years in the combination group and 57.1 ± 10.2 years in the monotherapy group, and the mean body mass index (BMI) was 30.5 ± 5.0 kg/m^2^ and 30.4 ± 4.8 kg/m^2^, respectively. Baseline HbA1c was 7.7% ± 1.3% (61 ± 14 mmol/mol) in the combination group and 7.6% ± 1.2% (60 ± 13 mmol/mol) in the monotherapy group and the baseline ALT levels were 81.5 ± 42.8 and 72.1 ± 30.7 U/L, respectively. FibroScan measurements using the M probe were available for a subset of participants (combination therapy group: *n* = 19; monotherapy group: *n* = 32).

**TABLE 1:** Baseline characteristics of the participants (full analysis set).

	Combination therapy (GLP‐1RA + SGLT2 inhibitor) (*n* = 24)	Monotherapy (GLP‐1RA alone) (*n* = 36)	*p*
Demographics			
Age (years)	56.0 ± 12.3	57.1 ± 10.2	0.719
Male sex, no. (%)	9 (37.5)	19 (52.8)	0.245
Body mass index (kg/m^2^)	30.5 ± 5.0	30.4 ± 4.8	0.912
Glycemic measures			
Fasting plasma glucose (mg/dL)	139.3 ± 27.4	142.1 ± 34.1	0.734
HbA1c, %	7.7 ± 1.3	7.6 ± 1.2	0.782
Liver‐related laboratory tests			
Aspartate aminotransferase (AST) (U/L)	57.0 ± 21.6	56.5 ± 25.2	0.937
Alanine aminotransferase (ALT) (U/L)	81.5 ± 42.8	72.1 ± 30.7	0.324
Gamma‐glutamyl transferase (GGT) (U/L)	111.0 ± 95.9	95.4 ± 100.5	0.437
Platelet count (×10^4^/μL)	21.0 ± 7.7	22.2 ± 7.8	0.558
Albumin (g/dL)	4.3 ± 0.3	4.2 ± 0.3	0.274
Lipids and renal function			
Total cholesterol (mg/dL)	204.9 ± 36.6	188.9 ± 34.3	0.090
Triglycerides (mg/dL)	162.1 ± 84.1	149.4 ± 72.3	0.536
LDL cholesterol (mg/dL)	118.1 ± 31.2	113.4 ± 32.6	0.580
HDL cholesterol (mg/dL)	48.6 ± 16.3	42.9 ± 9.4	0.089
Creatinine (mg/dL)	0.73 ± 0.20	0.68 ± 0.16	0.315
Baseline histological characteristics			
Fibrosis stage, no. (%)			0.657
Stage 1	4 (16.7)	10 (27.8)	
Stage 2	5 (20.8)	5 (13.9)	
Stage 3	8 (33.3)	9 (25.0)	
Stage 4	7 (29.2)	12 (33.3)	
NAFLD activity score (0–8)	6.0 ± 1.1	6.0 ± 1.4	0.356*
Steatosis score (0–3)	2.46 ± 0.59	2.42 ± 0.65	0.805*
Lobular inflammation score (0–3)	2.08 ± 0.65	2.08 ± 0.65	1.000
Ballooning score (0–2)	1.42 ± 0.50	1.47 ± 0.51	0.672
FibroScan (subset)			
Liver stiffness (kPa) (M probe)	11.7 ± 7.8 (*n* = 19)	11.2 ± 5.5 (*n* = 32)	0.770
Controlled attenuation parameter (CAP) (dB/m) (M probe)	312.4 ± 35.5 (*n* = 19)	328.3 ± 38.5 (*n* = 32)	0.149

*Note:* Data are mean ± SD or no. (%).

*p* values were obtained using the chi‐square tests for categorical variables and two‐sample *t*‐tests for continuous variables.

Histological scores (NAFLD activity score and component scores) are summarised as mean ± SD, derived from the observed category distributions.

FibroScan assessments (M probe) were available for a subset of participants; missing assessments were primarily owing to obesity‐related limitations.

* *p* values should be interpreted with caution owing to small expected cell counts.

### 3.3 Primary Histological Endpoints (FAS With Multiple Imputations)

Liver biopsy specimens from 58 participants (combination therapy group: *n* = 22; monotherapy: *n* = 36) were evaluated at Week 52. Two participants in the combination group declined repeat biopsy due to pain during the baseline biopsy; their Week‐52 histological outcomes were treated as missing and handled using multiple imputations in the pre‐specified primary analysis of the FAS (24 vs. 36).

At Week 52, all three pre‐specified histological endpoints numerically favoured combination therapy over monotherapy (Figure 2, Table S1). Resolution of MASH without worsening of fibrosis occurred in an estimated 34.9% of participants in the combination group and 19.4% in the monotherapy group (adjusted risk difference, 15.0 percentage points; 95% CI, −8.6 to 38.6; *p* = 0.214). A ≥ 1‐point improvement in NAS without worsening fibrosis was observed in 75.5% and 55.6% of participants, respectively (adjusted risk difference, 21.1 percentage points; 95% CI, −1.2 to 43.4; *p* = 0.063). Improvement in fibrosis stage by ≥ 1 without worsening of MASH occurred in 26.9% and 13.9%, respectively (adjusted risk difference, 15.2 percentage points; 95% CI, −4.9 to 35.4; *p* = 0.139). Although none of the pre‐specified comparisons reached statistical significance, point estimates consistently favoured combination therapy, with the largest signal observed for improvement in NAS without worsening fibrosis.

**FIGURE 2:**
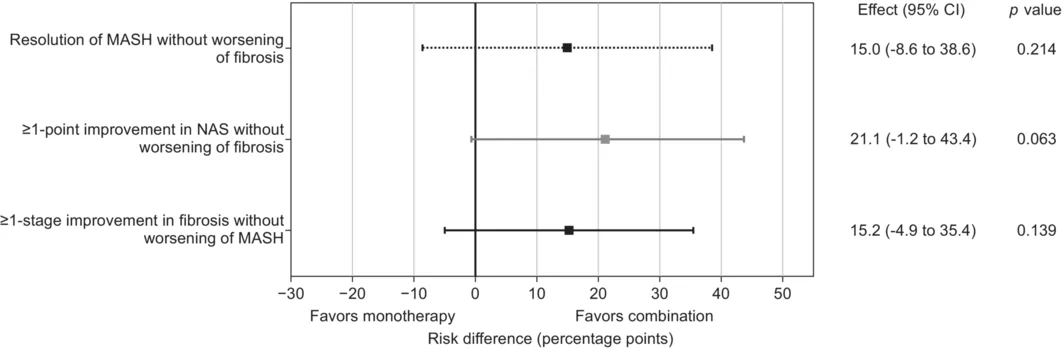
Primary histological endpoints at Week 52 (full analysis set with multiple imputations). Shown are the adjusted risk differences (percentage points) and 95% confidence intervals for the pre‐specified primary histological endpoints comparing combination therapy with glucagon‐like peptide‐1 receptor agonist (GLP‐1RA) plus sodium–glucose cotransporter 2 inhibitor versus GLP‐1RA monotherapy (full analysis set: *n* = 24 and *n* = 36, respectively). Missing Week‐52 biopsy outcomes were handled using multiple imputations. Treatment‐group comparisons were performed using a Cochran–Mantel–Haenszel approach, adjusting for baseline fibrosis stage (1–4) and baseline NAFLD activity score categories (4–6 and 7–8).

### 3.4 Pre‐Specified Per‐Protocol Analysis

In the PPS analysis restricted to participants with evaluable Week‐52 liver biopsy specimens (combination therapy group: *n* = 22; monotherapy group: *n* = 36), the results were directionally consistent with those of the primary analysis (Figure S1 and Table S2). Resolution of MASH without worsening of fibrosis occurred in 36.4% of participants in the combination group and 19.4% in the monotherapy group (adjusted risk difference, 16.7 percentage points; 95% CI, −7.3 to 40.8; *p* = 0.173). A ≥ 1‐point improvement in NAS without worsening of fibrosis was observed in 77.3% and 55.6% of participants, respectively (adjusted risk difference, 22.8 percentage points; 95% CI, 0.4 to 45.3; *p* = 0.046). Improvement in fibrosis stage by ≥ 1 without worsening MASH occurred in 27.3% and 13.9% of participants, respectively (adjusted risk difference, 15.1 percentage points; 95% CI, −5.3 to 35.5; *p* = 0.146). The between‐group difference in improvement in NAS without worsening of fibrosis was nominally significant in the PPS analysis.

Descriptive analyses of histological components demonstrated reductions in NAS and improvements in steatosis, lobular inflammation and hepatocellular ballooning in both treatment groups, whereas changes in fibrosis stage were limited (Table S3).

### 3.5 Secondary Outcomes

Secondary outcomes were analysed using the PPS; sample sizes varied according to outcome‐specific data availability (Figure S2 and Table 2).

**TABLE 2:** Key secondary outcomes at Week 52.

Outcome	Combination (*n*)	Adjusted change, LS mean (SE)	Monotherapy (*n*)	Adjusted change mean (SE)	Adjusted change, LS mean (SE)	*p*
Body weight (% change)	24	−8.9 (1.0)	36	−5.2 (0.8)	−3.7 (−6.0 to −1.4)	0.002
HbA1c (percentage points)	24	−1.6 (0.2)	36	−1.0 (0.1)	−0.6 (−0.9 to −0.2)	0.003
AST (U/L)	24	−28.8 (2.9)	36	−18.9 (2.4)	−9.8 (−16.8 to −2.8)	0.007
ALT (U/L)	24	−42.7 (4.1)	36	−28.3 (3.3)	−14.4 (−24.3 to −4.5)	0.005
FibroScan liver stiffness (% change; M probe)	17	−30.7 (6.2)	30	−11.4 (4.7)	−19.3 (−33.8 to −4.8)	0.010
FibroScan CAP (% change; M probe)	17	−11.4 (4.0)	30	−9.5 (3.0)	−2.0 (−11.8 to 7.9)	0.691

*Note:* Values represent adjusted changes from baseline, presented as least‐squares mean (SE) unless otherwise indicated.

Adjusted changes and between‐group differences were estimated using analysis of covariance, including treatment group, baseline value of the outcome, baseline fibrosis stage (1–4) and baseline NAFLD activity score category (4–6 vs. 7–8). The effect measure is the adjusted between‐group difference in change (combination therapy minus monotherapy), with negative values indicating a greater reduction in the combination group.

FibroScan analyses were performed on participants with available M‐probe measurements; missing measurements were primarily due to obesity‐related limitations. *p* values are two‐sided and were not adjusted for multiple comparisons.

Abbreviations: ALT, alanine aminotransferase; AST, aspartate aminotransferase; CAP, controlled attenuation parameter; HbA1c, glycated haemoglobin.

### 3.6 Fibrosis Category Change

When fibrosis stage was categorised as improved, unchanged or worsened at Week 52, improvement was observed in 27.3% (6/22) of participants in the combination group and 16.7% (6/36) in the monotherapy group (*p* = 0.219), whereas fibrosis worsened in 18.2% (4/22) and 27.8% (10/36), respectively (*p* = 0.259).

### 3.7 Body Weight and Glycemic Control

The combination group demonstrated a greater percent reduction in body weight from baseline to Week 52 than did the monotherapy group (adjusted mean difference, −3.7%; 95% CI, −6.0 to −1.4; *p* = 0.002). Glycemic control also improved more with combination therapy: HbA1c decreased by an adjusted mean of 1.6 percentage points in the combination group versus 1.0 percentage points in the monotherapy group (adjusted mean difference, −0.6 percentage points; 95% CI, −0.9 to −0.2; *p* = 0.003; Figure S2 and Table 2). Longitudinal analyses in the PPS showed that body weight and HbA1c levels declined rapidly by Week 13 and remained lower through Week 52 in the combination group than in the monotherapy group (Figure 3A,B).

**FIGURE 3:**
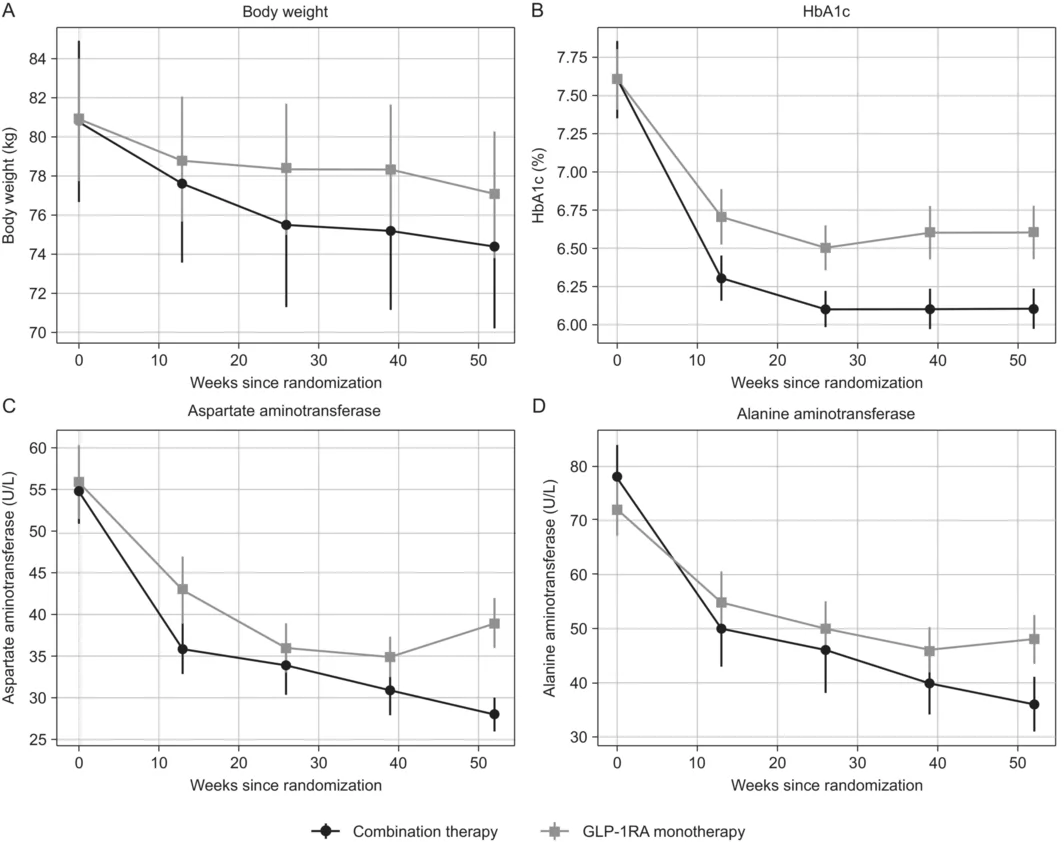
Changes in body weight, HbA1c and liver enzymes over time (per‐protocol set). Mean values with standard errors are shown for (A) body weight, (B) glycated haemoglobin, (C) aspartate aminotransferase and (D) alanine aminotransferase from baseline to Week 52 in the combination therapy (glucagon‐like peptide‐1 receptor agonist [GLP‐1RA] plus sodium–glucose cotransporter 2 inhibitor) and GLP‐1RA monotherapy groups. Data are shown for participants in the per‐protocol set with measurements available for each visit. Longitudinal changes were evaluated using mixed models for repeated measures, as described in Section 2, with values representing observed means ± standard errors at each visit.

### 3.8 Liver Enzymes and FibroScan

AST and ALT levels decreased to a greater extent in the combination group. The adjusted between‐group differences in change from baseline to Week 52 were −9.8 U/L for AST (95% CI, −16.8 to −2.8; *p* = 0.007) and −14.4 U/L for ALT (95% CI, −24.3 to −4.5; *p* = 0.005; Table 2). In longitudinal analyses based on the PPS, AST and ALT levels declined rapidly by Week 13 and remained lower through Week 52 in the combination group than in the monotherapy group (Figure 3C,D).

FibroScan‐derived liver stiffness, assessed with the M probe, showed a greater percent reduction in the combination group than in the monotherapy group (adjusted mean difference, −19.3 percentage points; 95% CI, −33.8 to −4.8; *p* = 0.010), whereas the between‐group difference in CAP was not significant (adjusted mean difference, −2.0 percentage points; 95% CI, −11.8 to 7.9; *p* = 0.691). FibroScan analyses were performed among participants with available measurements; missing measurements were primarily due to obesity‐related technical limitations.

### 3.9 Exploratory Post Hoc Analysis According to Luseogliflozin Dose Escalation

An exploratory post hoc analysis was performed within the combination therapy group according to luseogliflozin dose‐escalation status (Tables S4 and S5). Baseline clinical and histological characteristics were broadly comparable between participants maintained on 2.5 mg and those whose dose was escalated to 5 mg. No clear differences were observed in histological outcomes, body weight reduction, glycemic control or liver enzyme improvement according to the dose‐escalation status.

### 3.10 Safety

Adverse events occurred in 16 of 24 participants (66.7%) in the combination therapy group and 19 of 36 (52.8%) in the monotherapy group (Table S6). The most frequently reported events were gastrointestinal in nature. Nausea occurred in five participants (20.8%) in the combination group and 12 participants (33.3%) in the monotherapy group, while diarrhoea occurred in three (12.5%) and 10 (27.8%) participants, respectively. Vomiting was reported in one participant (4.2%) in the combination group and four participants (11.1%) in the monotherapy group. Hypoglycemia was infrequent (1 [4.2%] vs. 1 [2.8%]). No serious adverse events, deaths or treatment discontinuations due to adverse events were reported. The only discontinuations were due to withdrawal of consent; two participants in the combination group declined a repeat liver biopsy because of pain during the baseline procedure.

## 4 Discussion

In this randomised trial involving adults with biopsy‐proven MASH and type 2 diabetes mellitus, the addition of an SGLT2 inhibitor (luseogliflozin) to GLP‐1RA therapy for 52 weeks resulted in numerically favourable trends across the pre‐specified histological endpoints compared with GLP‐1RA monotherapy; however, the pre‐specified primary analysis in the FAS with multiple imputations did not demonstrate statistically significant differences. In the pre‐specified per‐protocol analysis, a nominal improvement in the NAS without worsening of fibrosis was observed. Combination therapy also resulted in significant improvements in body weight, HbA1c, aminotransferase levels and liver stiffness, as assessed by FibroScan. The safety findings were consistent with known class effects, with no serious adverse events, deaths or discontinuations due to adverse events.

Our findings extend prior evidence supporting incretin‐based therapy and SGLT2 inhibition in MASLD with type 2 diabetes mellitus. GLP‐1RAs have shown histological benefits in biopsy‐based trials, particularly for steatohepatitis activity, whereas fibrosis effects have been less consistent [8, 9, 10, 19]. SGLT2 inhibitors have also been associated with improvements in liver enzymes and steatosis‐ and fibrosis‐related measurements. Biopsy‐based randomised studies from Japan support their potential hepatic benefit, although between‐group differences have been modest and inconsistent [11, 12, 14]. Recent evidence, including a pooled meta‐analysis of luseogliflozin and a large nationwide study of SGLT2 inhibitors, has further demonstrated improvements in hepatic steatosis, fibrosis‐related indices and liver‐related outcomes in patients with type 2 diabetes and MASLD. These findings are consistent with the improvements in aminotransferase levels and FibroScan‐derived liver stiffness observed in our trial [20, 21].

However, most prior studies have evaluated GLP‐1RAs or SGLT2 inhibitors as a monotherapy (or against alternative glucose‐lowering agents) rather than testing the incremental effects of adding an SGLT2 inhibitor to GLP‐1RA therapy. Evidence for GLP‐1RA–SGLT2 inhibitor combination effects on MASLD/MASH therefore remains limited. A randomised pilot study (EXENDA) reported that exenatide plus dapagliflozin did not produce an additive reduction in hepatocellular lipid content compared with dapagliflozin alone, despite greater glycemic improvement. This highlights the difficulty of demonstrating additivity when relying primarily on liver fat‐based endpoints over a short duration [22].

Observational and real‐world studies have suggested potential liver‐related benefits of GLP‐1RA–SGLT2 inhibitor combination therapy, including lower risks of adverse liver outcomes, reduced incidence of NAFLD and improvements in non‐invasive markers such as the fibrosis‐4 (FIB‐4) index [23, 24, 25]. However, these studies are limited by residual confounding factors and reliance on surrogate rather than histological endpoints. Our randomised trial with paired liver biopsies was designed to address this gap by directly testing whether adding an SGLT2 inhibitor to GLP‐1RA therapy at standard antidiabetic doses provides incremental histological benefit in biopsy‐proven MASH.

The observed pattern of effects is biologically plausible, given the complementary mechanisms of GLP‐1RAs and SGLT2 inhibitors. GLP‐1RAs reduce energy intake and body weight and improve insulin sensitivity, which are thought to decrease hepatic de novo lipogenesis and lipotoxic stress, thereby improving steatohepatitis activity [8, 9, 10]. SGLT2 inhibitors lower glucotoxicity via urinary glucose excretion and induce a sustained negative energy balance with reduction in visceral adiposity, which may contribute to a decrease in hepatic fat flux and oxidative stress [11, 14]. These partially distinct pathways could plausibly yield additive improvements in weight and glycemia, as reflected in our trial by larger reductions in body weight and HbA1c levels, and translate into downstream improvements in aminotransferases and liver stiffness.

In patients with MASH and type 2 diabetes mellitus, selecting therapies with complementary metabolic mechanisms, such as GLP‐1RAs and SGLT2 inhibitors, may offer a practical opportunity to modify liver disease activity while simultaneously improving glycemic control and cardiometabolic risk [26, 27]. As semaglutide has recently been approved by the FDA for noncirrhotic MASH, integrating liver‐focused considerations into daily diabetes mellitus treatment decisions may represent a pragmatic approach for this high‐risk population.

This study had some limitations. The sample size was modest, and probabilistic minimisation resulted in unequal group sizes despite the approximately balanced allocation assumed in the original sample size calculation. Together with the smaller number of evaluable Week‐52 biopsies in the combination therapy group, this imbalance reduced the statistical power to detect between‐group differences in the primary histological endpoints. To address potential bias associated with missing Week‐52 histological data, the primary analysis was performed in the full analysis set using multiple imputation, and a pre‐specified per‐protocol analysis was also conducted. FibroScan data were available only in a subset of participants and may be subject to technical limitations, particularly in individuals with higher BMI; therefore, analyses were restricted to participants with valid and reliable measurements. The 52‐week duration precluded the assessment of long‐term clinical outcomes. Additionally, the study was conducted in Japan in a population with a mean BMI of approximately 30 kg/m^2^, which may limit generalizability.

Future studies with larger sample sizes, longer follow‐up, standardised elastography protocols including appropriate probe selection and more diverse populations are warranted to confirm these findings and to evaluate long‐term clinical outcomes. Larger, multicenter trials powered by histological and clinical outcomes are warranted to confirm the additive benefits and define the optimal role of GLP‐1RA–SGLT2 inhibitor combination therapy in type 2 diabetes mellitus and comorbidity of MASH.

## 5 Conclusion

In adults with biopsy‐proven MASH and type 2 diabetes mellitus, adding an SGLT2 inhibitor to GLP‐1RA therapy at standard antidiabetic doses did not significantly improve the pre‐specified histological endpoints in the FAS analysis. Combination therapy was associated with significant improvements in body weight, glycemic control, aminotransferase levels and liver stiffness without any new safety concerns.

## Author Contributions

T.M. conceived and designed the study, supervised the clinical trial as the principal investigator, interpreted the data and wrote the manuscript. O.Y. contributed to the study design and coordinated clinical research operations. S.F. developed the statistical analysis plan, supervised and verified the statistical analyses, had access to the analytical dataset and confirmed the accuracy of the analyses. Statistical analyses were conducted in collaboration with an independent statistical support organisation under the supervision of S.F. M.H. contributed to data management. M.A. and Y.T. performed the histopathological assessment. A.K., T.W., Y.O., A.Y., Y.N. and T.K. contributed to patient enrollment, clinical data collection and study conduct. E.T. contributed to patient registration and allocation. R.Y. contributed to study coordination. Y.H. supervised the overall study and critically revised the manuscript for important intellectual content. All authors reviewed and approved the final version of the manuscript.

## Funding

This work was supported by Taisho Toyama Pharmaceutical Company.

## Conflicts of Interest

Y.H. reports receiving a research grant from Taisho Pharmaceutical Co. Ltd. related to this study and lecture fees from Taisho Pharmaceutical Co. Ltd. and Kowa Company Ltd. T.M. reports receiving lecture fees related to MASLD and diabetes mellitus from Taisho Pharmaceutical Co. Ltd., Novo Nordisk Pharma Ltd., Eli Lilly Japan K.K., Boehringer Ingelheim Japan Inc., Sumitomo Pharma Co. Ltd., Tanabe Pharma Corporation and Kowa Company Ltd. S.F. reports receiving lecture fees related to diabetes mellitus from Taisho Pharmaceutical Co. Ltd., Novo Nordisk Pharma Ltd., Eli Lilly Japan K.K., Sumitomo Pharma Co. Ltd., Tanabe Pharma Corporation and Kowa Company Ltd. The other authors declare no conflicts of interest.

## Supporting information


**Table S1:** Primary histological endpoints at Week 52 (full analysis set with multiple imputations).
**Table S2:** Primary histological endpoints at Week 52 (per‐protocol set).
**Table S3:** Within‐group changes in histological components from baseline to Week 52 (per‐protocol set).
**Table S4:** Baseline characteristics according to luseogliflozin dose escalation within the combination‐therapy group.
**Table S5:** Exploratory post hoc subgroup analysis according to luseogliflozin dose escalation within the combination‐therapy group.
**Table S6:** Adverse events (safety analysis set).

**Figure S1:** Primary histological endpoints at Week 52 in the per‐protocol set.

**Figure S2:** Key secondary outcomes at Week 52.

## Data Availability

Data are not publicly available due to institutional and ethical restrictions. Access to de‐identified data may be granted on a case‐by‐case basis upon reasonable request, subject to institutional approval and a formal data use agreement.
