# Supplementary material for: Efficacy of Luseogliflozin Added to Semaglutide in Patients With Metabolic Dysfunction‐Associated Steatohepatitis and Type 2 Diabetes Mellitus: A Randomised, Open‐Label Trial
Source: Diabetes Obes Metab. 2026 Aug 17;28(11):10163–72. doi: 10.1111/dom.71233 (PMC13646358; doi:10.1111/dom.71233)
Supplement: Supplementary file 1 [file DOM-28-10163-s002.pdf]

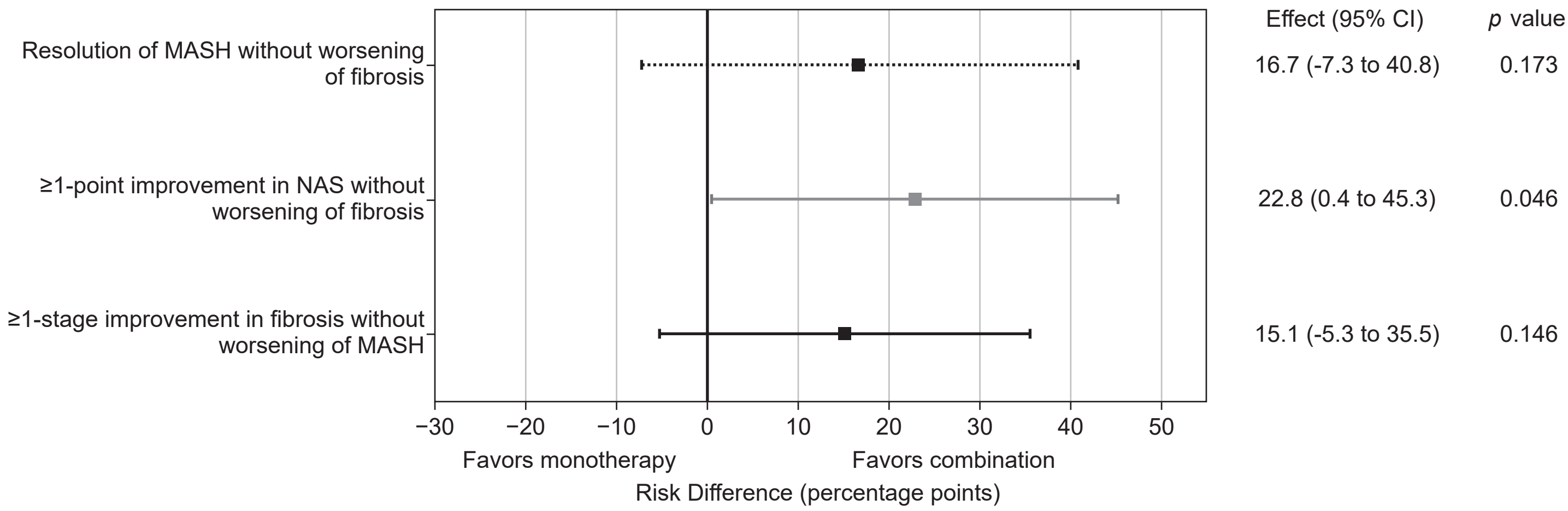
Effect (95% CI)
p value
Resolution of MASH without worsening of fibrosis
16.7 (-7.3 to 40.8)
0.173
≥1-point improvement in NAS without worsening of fibrosis
22.8 (0.4 to 45.3)
0.046
≥1-stage improvement in fibrosis without worsening of MASH
15.1 (-5.3 to 35.5)
0.146
−30
−20
−10
0
10
20
30
40
50
Favors monotherapy
Favors combination
Risk Difference (percentage points)
