# Supplementary material for: Efficacy of Luseogliflozin Added to Semaglutide in Patients With Metabolic Dysfunction‐Associated Steatohepatitis and Type 2 Diabetes Mellitus: A Randomised, Open‐Label Trial
Source: Diabetes Obes Metab. 2026 Aug 17;28(11):10163–72. doi: 10.1111/dom.71233 (PMC13646358; doi:10.1111/dom.71233)
Supplement: Supplementary file 2 [file DOM-28-10163-s003.pdf]

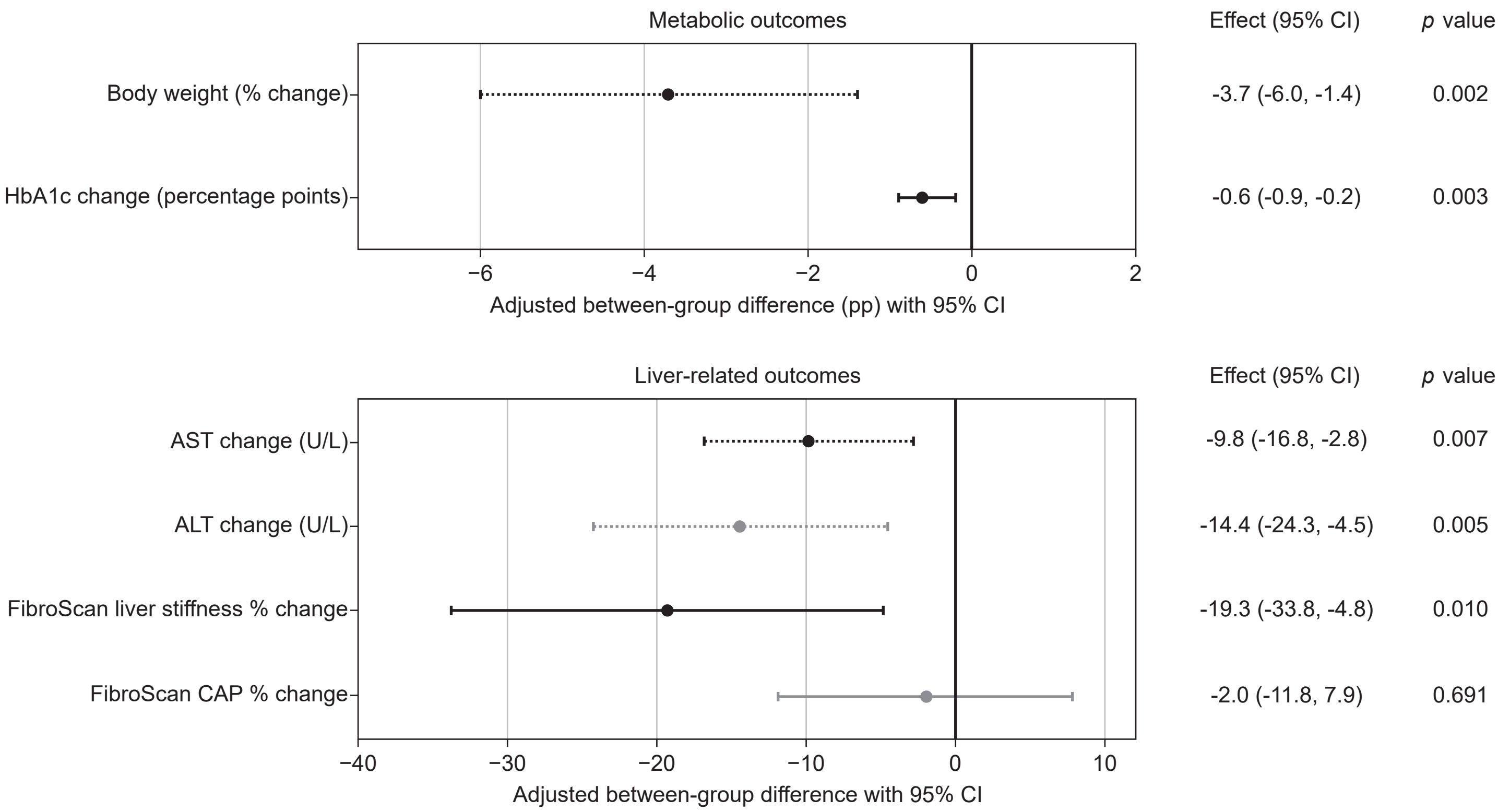
Metabolic outcomes
Effect (95% CI)
p value
Body weight (% change)
-3.7 (-6.0, -1.4)
0.002
HbA1c change (percentage points)
-0.6 (-0.9, -0.2)
0.003
−6
−4
−2
0
2
Adjusted between-group difference (pp) with 95% CI
Liver-related outcomes
Effect (95% CI)
p value
AST change (U/L)
-9.8 (-16.8, -2.8)
0.007
ALT change (U/L)
-14.4 (-24.3, -4.5)
0.005
FibroScan liver stiffness % change
-19.3 (-33.8, -4.8)
0.010
FibroScan CAP % change
-2.0 (-11.8, 7.9)
0.691
−40
−30
−20
−10
0
10
Adjusted between-group difference with 95% CI
