# Supplementary material for: Efficacy of Luseogliflozin Added to Semaglutide in Patients With Metabolic Dysfunction‐Associated Steatohepatitis and Type 2 Diabetes Mellitus: A Randomised, Open‐Label Trial
Source: Diabetes Obes Metab. 2026 Aug 17;28(11):10163–72. doi: 10.1111/dom.71233 (PMC13646358; doi:10.1111/dom.71233)
Supplement: Supplementary file 3 [file DOM-28-10163-s001.docx]

**Electronic Supplementary Material**

**Supplemental Table S1. Primary Histological Endpoints at Week 52 (Full Analysis Set with Multiple Imputations)**

| Endpoint | Combination therapy  (n=24),  Estimated % | Monotherapy  (n=36),  Estimated % | Adjusted risk difference,  pp (95% CI) | *p* value |
| --- | --- | --- | --- | --- |
| Resolution of MASH without worsening of fibrosis | 34.9 | 19.4 | 15.0 (−8.6 to 38.6) | 0.214 |
| ≥1-point improvement in NAS without worsening of fibrosis | 75.5 | 55.6 | 21.1 (−1.2 to 43.4) | 0.063 |
| ≥1-stage improvement in fibrosis without worsening of MASH | 26.9 | 13.9 | 15.2 (−4.9 to 35.4) | 0.139 |

Data are estimated as percentages pooled across multiple imputations unless otherwise indicated.

The full analysis set included all randomized participants (n=60).

Week-52 liver-biopsy specimens were evaluable in 58 participants (22 in the combination therapy group and 36 in the monotherapy group); missing week-52 histological outcomes were handled using multiple imputations, with estimates combined across 200 imputed datasets.

Adjusted risk differences (percentage points; combination minus monotherapy) and 95% confidence intervals (CIs) were estimated using the Cochran–Mantel–Haenszel method, stratified by baseline fibrosis stage [1–4] and baseline NAFLD activity score (4–6 vs. 7–8). *p* values were calculated using the same method.

MASH, metabolic dysfunction–associated steatohepatitis; NAS, NAFLD activity score; pp, percentage points.

**Supplemental Table S2. Primary Histological Endpoints at Week 52 (Per-protocol Set)**

| Endpoint | Combination therapy (n=22) no. (%) | Monotherapy (n=36) no. (%) | Adjusted risk difference, pp (95% CI) | *p* value |
| --- | --- | --- | --- | --- |
| Resolution of MASH without worsening of fibrosis | 8 (36.4) | 7 (19.4) | 16.7 (−7.3 to 40.8) | 0.173 |
| ≥1-point improvement in NAS without worsening of fibrosis | 17 (77.3) | 20 (55.6) | 22.8 (0.4 to 45.3) | 0.046 |
| ≥1-stage improvement in fibrosis without worsening of MASH | 6 (27.3) | 5 (13.9) | 15.1 (−5.3 to 35.5) | 0.146 |

Data are presented as the number of participants (%), unless otherwise indicated.

The per-protocol set included participants with evaluable week-52 liver-biopsy specimens (22 in the combination therapy group and 36 in the monotherapy group).

Adjusted risk differences (percentage points; combination minus monotherapy) and 95% confidence intervals (CIs) were estimated using the Cochran–Mantel–Haenszel method, stratified by baseline fibrosis stage [1–4] and baseline NAFLD activity score (4–6 vs. 7–8). *p* values were calculated using the same method.

MASH, metabolic dysfunction–associated steatohepatitis; NAS, NAFLD activity score; pp, percentage points.

**Supplemental Table S3. Within-Group Changes in Histological Components from Baseline to Week 52 (Per-protocol Set)**

| Outcome | Combination therapy (n=22) | Monotherapy (n=36) |
| --- | --- | --- |
| NAFLD activity score (total) | −2.5 ± 1.8 (median −2.5) | −1.9 ± 1.8 (median −2.0) |
| Steatosis score | −1.0 ± 0.8 (median −1.0) | −0.7 ± 0.8 (median −0.5) |
| Lobular inflammation score | −0.7 ± 0.9 (median −1.0) | −0.7 ± 0.7 (median −1.0) |
| Hepatocellular ballooning score | −0.9 ± 0.6 (median −1.0) | −0.6 ± 0.8 (median −1.0) |
| Fibrosis stage | −0.2 ± 0.9 (median 0.0) | 0.1 ± 1.0 (median 0.0) |

Values are presented as mean±SD (median). Negative values indicate improvement from baseline.

The per-protocol set included patients with evaluable liver biopsy specimens collected at week 52.

Changes represent within-group differences from baseline to week 52. No formal hypothesis testing was performed for these within-group comparisons; analyses were descriptive and exploratory.

**Supplemental Table S4. Baseline Characteristics According to Luseogliflozin Dose Escalation Within the Combination-Therapy Group**

| Characteristic | Maintained at 2.5 mg (n=15) | Escalated to 5 mg (n=9) | *p* value |
| --- | --- | --- | --- |
| Age, years | 56.3±12.3 | 55.4±13.0 | 0.858 |
| Body mass index, kg/m² | 29.9±4.6 | 31.7±5.6 | 0.512 |
| HbA1c, % | 7.71±1.15 | 7.66±1.55 | 0.590 |
| AST, U/L | 58.3±22.9 | 54.8±20.4 | 0.788 |
| ALT, U/L | 77.1±41.7 | 88.8±46.2 | 0.512 |
| Fibrosis stage | 2.80±1.01 | 2.67±1.22 | 0.853 |
| NAFLD activity score | 5.87±1.19 | 6.11±1.05 | 0.689 |

Values are presented as mean±SD.

Participants who underwent dose escalation were selected during follow-up because of an insufficient treatment response; therefore, comparisons between dose subgroups are exploratory and potentially subject to confounding by indication.

HbA1c, glycated hemoglobin; AST, aspartate aminotransferase; ALT, alanine aminotransferase.

**Supplemental Table S5. Exploratory Post Hoc Subgroup Analysis According to Luseogliflozin Dose Escalation Within the Combination-Therapy Group**

| Outcome | Maintained at 2.5 mg (n=15) | Escalated to 5 mg (n=9) | *p* value |
| --- | --- | --- | --- |
| NAS change from baseline | −2.43±1.70 | −2.75±2.05 | 0.627 |
| Fibrosis stage change from baseline | −0.07±0.62 | −0.38±1.30 | 0.763 |
| Body weight change (%) | −8.71±3.83 | −8.18±5.27 | 0.633 |
| HbA1c change (percentage points) | −1.67±1.13 | −1.53±1.12 | 0.720 |
| AST change (U/L) | −27.6±16.4 | −28.4±15.4 | 0.929 |
| ALT change (U/L) | −37.5±23.0 | −50.8±24.7 | 0.210 |
|  |  |  |  |
| Histological endpoint | Maintained at 2.5 mg (n=14) | Escalated to 5 mg (n=8) | *p* value |
| Resolution of MASH without worsening of fibrosis | 4 (28.6%) | 4 (50.0%) | 0.386 |
| ≥1-point improvement in NAS without worsening of fibrosis | 11 (78.6%) | 6 (75.0%) | 1.000 |
| ≥1-stage improvement in fibrosis without worsening of MASH | 3 (21.4%) | 3 (37.5%) | 0.624 |

Values are presented as mean±SD or n (%).

Continuous variables were compared using the Wilcoxon rank-sum test and categorical variables using the Fisher's exact test. Since dose escalation was not randomized but determined according to treatment response, these exploratory analyses are potentially subject to confounding by indication and should not be interpreted as estimates of the efficacy of the 5 mg dose.

NAS, NAFLD activity score; MASH, metabolic dysfunction-associated steatohepatitis; HbA1c, glycated hemoglobin; AST, aspartate aminotransferase; ALT, alanine aminotransferase.

**Supplemental Table S6. Adverse Events (Safety Analysis Set)**

| Event | Combination therapy  (n=24)  no. (%) | Monotherapy  (n=36)  no. (%) |
| --- | --- | --- |
| Any adverse event | 16 (66.7) | 19 (52.8) |
| Nausea | 5 (20.8) | 12 (33.3) |
| Gastroesophageal reflux disease | 1 (4.2) | 2 (5.6) |
| Diarrhea | 3 (12.5) | 10 (27.8) |
| Dyspepsia | 0 (0.0) | 1 (2.8) |
| Decreased appetite | 1 (4.2) | 1 (2.8) |
| Injection-site reaction | 1 (4.2) | 0 (0.0) |
| Hypoglycemia | 1 (4.2) | 1 (2.8) |
| Fatigue | 5 (20.8) | 4 (11.1) |
| Abdominal pain | 1 (4.2) | 0 (0.0) |
| Abdominal discomfort | 0 (0.0) | 2 (5.6) |
| Abdominal distension | 2 (8.3) | 0 (0.0) |
| Constipation | 3 (12.5) | 5 (13.9) |
| Vomiting | 1 (4.2) | 4 (11.1) |
| Cystitis | 1 (4.2) | 0 (0.0) |
| Serious adverse event | 0 | 0 |
| Discontinuation due to adverse event | 0 | 0 |
| Death | 0 | 0 |

Data represent the number of participants experiencing at least one event (%). All events are listed regardless of causality. No serious adverse events, deaths, or discontinuations due to adverse events were observed.

**Supplemental Figure S1. Primary Histological Endpoints at Week 52 in the Per-protocol Set**

Adjusted risk differences (percentage points) and 95% confidence intervals are shown for the pre-specified primary histological endpoints comparing combination therapy with glucagon-like peptide-1 receptor agonist (GLP-1RA) plus sodium–glucose cotransporter 2 inhibitor versus GLP-1RA monotherapy among participants with evaluable week 52 liver biopsy specimens (per-protocol set; n=22 and n=36, respectively). Treatment-group comparisons were performed using the Cochran–Mantel–Haenszel approach, adjusted for baseline fibrosis stage [1–4] and baseline NAFLD activity score categories [4–8].

**Supplemental Figure S2. Key Secondary Outcomes at Week 52**

Adjusted between-group differences with 95% confidence intervals are shown for key secondary outcomes comparing combination therapy with glucagon-like peptide-1 receptor agonist (GLP-1RA) plus sodium–glucose cotransporter 2 inhibitor and GLP-1RA monotherapy. Analyses were conducted using available participants for each outcome, with no imputation unless otherwise specified. Adjusted treatment effects were estimated using analysis of covariance, adjusting for baseline fibrosis stage, baseline NAFLD activity score categories, and the corresponding baseline value for each outcome. FibroScan outcomes were analyzed in participants with available M-probe measurements; missing measurements were primarily due to obesity-related technical limitations.
